# Homocysteine, MTHFR C677T gene polymorphism, folic acid and vitamin B 12 in patients with retinal vein occlusion

**DOI:** 10.1186/1477-9560-3-13

**Published:** 2005-09-07

**Authors:** Paola Ferrazzi, Pierpaolo Di Micco, Ilaria Quaglia, Lisa Simona Rossi, Alessandro Giacco Bellatorre, Giorgio Gaspari, Lidia Luciana Rota, Corrado Lodigiani

**Affiliations:** 1 Thrombosis Center, Istituto Clinico Humanitas "IRCCS", Milan, Italy; 2 Ophtalmology Unit, Istituto Clinico Humanitas "IRCCS", Milan, Italy

**Keywords:** retinal vein occlusion, thrombophilia, homocysteine, MTHFR C677T, folic acid

## Abstract

**Background:**

Many available data have suggested that hyperhomocysteinaemia, an established independent risk factor for thrombosis (arterial and venous), may be associated with an increased risk of retinal vein occlusion (RVO).

**Aim of the study:**

To evaluate homocysteine metabolism in consecutive caucasian patients affected by RVO from Northern Italy.

**Patients and Methods:**

69 consecutive patients from Northern Italy (mean age 64.1 ± 14.6 yy) with recent RVO, were tested for plasma levels of homocysteine (tHcy: fasting and after loading with methionine), cyanocobalamine and folic acid levels (CMIA-Abbot) and looking for MTHFR C677T mutation (Light Cycler-Roche) and compared to 50 volunteers, enrolled as a control group.

**Results:**

Fasting levels of tHcy were significantly higher in patients than in controls: mean value 14.7 ± 7.7 vs 10.2 ± 8 nmol/ml. Post load levels were also significantly higher: mean value 42.7 ± 23.7 vs 30.4 ± 13.3 nmol/ml; Total homocysteine increase was also evaluated (i.e. Δ-tHcy) after methionine load and was also significantly higher in patients compared to control subjects: mean Δ-tHcy 27.8 ± 21.5 vs 21.0 ± 16 nmol/ml (normal value < 25 nmol/ml). Furthermore, patients affected by RVO show low folic acid and/or vitamin B12 levels, although differences with control group did not reach statistical significance. Heterozygous and homozygous MTHFR mutation were respectively in study group 46% and 29% vs control group 56% and 4%.

**Conclusion:**

our data confirm that hyperhomocysteinaemia is a risk factor for RVO, and also that TT genotype of MTHFR C677T is more frequently associated with RVO: if the mutation per se is a risk factor for RVO remains an open question to be confirmed because another study from US did not reveal this aspect.

Hyperomocysteinemia is modifiable risk factor for thrombotic diseases. Therefore, a screening for tHcy plasma levels in patients with recent retinal vein occlusion could allow to identify patients who might benefit from supplementation with vitamins and normalization of homocysteine levels, in fasting and after methionine load.

## Background

Retinal vein occlusion (RVO) is a multifactorial disease which may affect small, medium and large ocular vessels; central vein occlusion represents the most dangerous clinical entity [1]. However, RVO is considered an unusual site of thrombosis [2]. Pathogenesis of RVO may recognise a local disease such as glaucoma, ocular hypertension, optical neuropathy [3] and/or an underlying systemic disease such as hypertension, diabetes, atherosclerosis, dyslipidemia, hyperviscosity syndrome, local or systemic vasculytis [4-8]. Yet, also impairment of normal haemostasis with a trend toward hypercoagulable state has been described. Blandello et al. described increased levels of prothrombin fragment 1+2 and d-dimer [9] and Lijima et al. reported increased levels of thrombin-antithrombin complexes in subjects affected by RVO [10]. Inherited thrombophilia related to clotting inhibitors deficiency (i.e. protein C, Protein S and Antithrombin III deficiencies) [11,12] has been rarely reported such as clotting XII deficiency [13], while data on the role of factor V Leiden are still matter of discussion [14-18] and few data are available on the role of prothrombin A20210G gene polymorphism in RVO pathogenesis [17,19]. Acquired thrombophilia due to the presence of antiphospholipid syndrome (primary or secondary to immunopathological disease) is an established risk factor for RVO [20,21]. Also hypofibrinolysis is a thrombotic risk factor for RVO. Plasminogen deficiency and 4G/5G gene polymorphism of plasminogen activator inhibitor type 1, in fact, have been recently described as risk factors for RVO [22,23].

Homocysteine is a sulphur-containing amino acid, which results from the hydrolysis of S-adenosyl-homocysteine in the methionine metabolic cycle. Several condition may determine an increase of blood homocysteine such as an inadequate folate intake with diet, smoking, drugs (i.e. methotrexate, hormones, antiepileptic), renal failure and inherited gene polymorphism of methylene-tetra-hydro-folate-reductase (MTHFR). Increase in circulating homocysteine may trigger endothelial dysfunction through oxidative damage therefore inducing increased oxidation of low density lipoprotein, stimulation of smooth muscle cell proliferation and hypercoagulable state. Hyperhomocysteinemia has been reported as risk factor for arterial and/or venous thrombosis [24,25]. Yet, hyperhomocysteinaemia has also been described as risk factor for RVO [26], but data on large based population are still lacking.

The aim of this study is to evaluate the role of homocysteine metabolism in consecutive patients with RVO from Northern Italy.

## Patients and methods

### Patients

We selected 69 consecutive caucasian patients from Northern Italy (40 males and 29 females, mean age 64.1 ± 14.6 years) affected by retinal vein occlusion (RVO). RVO diagnosis was performed with fundus oculi examination and fluorangiography.

Thirtyeight/69 (55%) patients were affected by hypertension, 12/69 (17%) by diabetes, 18/69 (26%) by dyslipidemia, 4/69 (5%) by glaucoma, while 16/69 (23%) were smokers; none of them had been diagnosed for hyperviscosity syndrome or vasculitis.

Risk factors for RVO in all patients are summarised in table 1.

**Table 1:** Risk factor for RVO of study group and control subjects.

	**RVO subjects (69)**	**CG subjects (50)**	**P**
**Hypertension**	38 (55%)	15 (30%)	0.014
**Diabetes**	12 (17%)	9 (18%)	Ns
**Glaucoma**	6(8%)	0 (0%)	0.039
**Hyperviscosity syndrome**	0 (0%)	0 (0%)	Ns
**Vasculitis**	0 (0%)	0 (0%)	Ns
**Smoking**	16 (23%)	12 (24%)	Ns
**Dyslipidemia**	18 (26%)	12 (24%)	Ns

RVO: retinal vein occlusion

CG: control group

HYP: hypertension

DIAB: diabetes

SMOG: smoking

DYS: dyslipidemia

GLAU: glaucoma

HS: hyperviscosity syndrome

VASC: vasculytis

Ns: not significant

Anticardiolipin antibodies (IgM and IgG) anti-β2-glycoprotein I antibodies and lupus anticoagulant were assayed in all patients in order to detect antiphospholipid syndrome; so patients with antiphospholipid syndrome were excluded from the study. Furthermore, none patient showed kidney failure.

All patients agreed to enter in the study after an informed consent was obtained.

### Control group

As control group we selected 50 age-matched volunteers of the same ethnic background (38 males and 12 females, mean age 58.4 ± 12.2 years) without personal and familial history of thrombotic disorders (i.e. previous venous and/or arterial thrombosis). All subjects agreed to enter the study after an informed consent was obtained. Fifteen/50 (30%) subjects were affected by hypertension, 9/50 (18%) by diabetes, 12/50 (24%) by dyslipidemia, while 12/50 (24%) were smokers; none of them had been diagnosed for glaucoma, hyperviscosity syndrome or vasculitis. Risk factors for RVO of control subjects are also summarised in table 1.

#### Common risk factors for RVO

Common risk factors for RVO (i.e. hypertension, diabetes, smog, dyslipidemia, vasculitys, hyperviscosity syndrome, glaucoma) were evaluated by a thorough anamnesis, including questions concerning familial anamnesis, personal anamnesis and pharmacological anamnesis.

### Methods

Whole blood samples were collected from all subjects selected in the study by venipuncture from antecubital vein in order to screen possible involvement of homocysteine metabolism. Samples were collected nearly 5–7 days after diagnosis of RVO after an adequate treatment had been started.

All patients were assayed for plasma homocysteine (tHcy), fasting and after load with methionine, vitamin B 12 and folic acid levels and MTHFR C677T gene polymorphism.

#### First blood sample

The first blood sample was collected in EDTA to screen fasting homocysteine (FPIA-Abbot).

#### Second blood sample

Post-load homocysteine value was measured after methionine administration (3.8 g/m^2^) per os. Homocysteinaemia was tested on a blood sample collected after four hours, then evaluated by collection of a new blood sample in EDTA (FPIA-Abbot). No food containing methionine was allowed in the interval.

We evaluated total homocysteine increase after oral load of methyonine (i.e. Δ-tHcy) in the study group and in control subjects [27].

#### Third blood sample

The third blood sample was collected in SST II advanced tube in order to detect serum folic acid levels and serum vitamin B 12 levels (CMIA-Abbot).

#### Fourth blood sample

DNA was extracted using an automated procedure (MagNA PURE, Roche, Italy). Patients were screened for the C677T gene polymorphism of MTHFR using PCR amplification with specific primers and Light Cycler apparatus (Roche, Milan, Italy).

### Statistical analysis

Data are expressed as mean ± standard deviation (SD) or as number and percentage, as appropriate. Statistical analysis was performed with STATA 6 . Significance of differences was assessed by Student's t test for unpaired data, χ^2 ^test or Fisher exact test as appropriate; differences were considered to be significant if p < 0.05.

## Results

Common risk factors for RVO were evaluated and are summarised in table 1. Hypertension was present in 55% of patients compared to 30% of control group (p: 0.014); diabetes was present in 17% of patients compared to 18% of control group (p: ns); smoking was present in 23% of patients compared to 24% of control group (p: ns); dyslipidemia was present in 26% of patients compared to 24% of control group (p: ns); glaucoma was present in 8% of patients was absent in control subjects (p: 0.039); hyperviscosity syndrome and/or vasculytis were not found in study group nor in control subjects (p: ns).

Fasting levels of homocysteinaemia were significantly higher in study group than in control subjects (14.7 ± 9.9 vs 10.2 ± 8 nmol/ml, p: 0.02) (table 2).

**Table 2:** Data of homocysteine metabolism parameters of study group and control subjects.

**Test (unit of measurement)**	**RVO subjects (69)**	**GC subjects (50)**	**P**
**Hcy (nmol/ml)**	14.7 ± 9.9	10.2 ± 8	0.02
**PL-Hcy (nmol/ml)**	42.7 ± 23.7	30.4 ± 13.3	<0.01
**Δ-tHcy (nmol/ml)**	27.8 ± 21.5	21.0 ± 16.0	<0.01
**Serum folic acid (ng/ml)**	6.27 ± 3.8	7.22 ± 3.5	Ns
**Serum B12 vit. (pg/ml)**	403.3 ± 202.0	601.6 ± 145.4	Ns
**MTHFR C677T heterozigosity (%)**	46,7	56,25	Ns
**MTHFR C677T homozigosity (%)**	29,3	4,17	<0.01

Hcy: homocysteine

PL-Hcy, post load homocysteine

Δ-tHcy: total homocysteine increase

MTHFR: methylene tetra hydrofolate reductase

RVO: retinal vein occlusion

CG: control group

Ns: not significant

Post-load levels of homocysteine (PL-Hcy) were also significantly higher in study group than in control group (42.7 ± 23.7 vs 30.4 ± 13.3 nmol/ml, p: < 0.01; normal value < 38 nmol/ml), as far as total homocysteine increase (Δ-tHcy) (27.8 ± 21.5 vs 21.0 ± 16 nmol/ml, p: < 0.01; normal value < 25 nmol/ml) (table 2).

Δ-tHcy increase after methionine load was significantly higher in study group than in control subjects (mean Δ-tHcy 27.8 ± 21.5 vs 19.8 ± 16 nmol/ml, p: < 0.01; normal value < 25 nmol/ml) (table 2).

Serum folic acids levels were 6.27 ± 3.8 ng/ml in patients vs 7.22 ± 3.5 ng/ml in control group (p: ns) (table 2), while serum vitamin B 12 levels were 403.3 ± 202.1 pg/ml in patients vs 512.6 ± 144.2 pg/ml in control subjects (p: ns) (table 2). Three/69 (4%) patients of study group showed low vitamin B 12 levels (i.e. < 150 pg/ml) vs none in the control group, while 11/69 (15%) of patients showed low folic acids levels (i.e. < 3 ng/ml) vs 2/50 (2.5%) in the control group. Among these, six patients with low folic acid and/or vitamin B 12 serum levels showed high fasting Hcy levels.

MTHFR C677T gene polymorphism was searched in 63/69 patients (91%) and was found in 75% of patients. 29/63 (46%) showed heterozigosity for MTHFR C677T gene polymorphism, while 18/63 (29%) had homozigosity for MTHFR C677T gene polymorphism. 48/50 subjects in the control group underwent genetic test to detect MTHFR C677T gene polymorphism: 27/48 (56%) showed heterozygous mutation (p: ns), while 2/48 (4%) showed homozygous mutation (p: 0.01) (table 2).

## Discussion

RVO is a multifactorial disease which includes also retinal vein thrombosis and its pathophysiology may be due to local and/or systemic risk factor [1]. We may recognise several types of RVO depending on the site of occlusion (branch RVO, central RVO, hemicentral RVO) and for localisation in large, medium or small-calibre veins; from a clinical point of view RVO is usually associated with visual loss of variable degree. An association of RVO with impairment of haemostasis with a trend toward hypercoagulable state has frequently been ruled out in case of retinal vein thrombosis by several reports, but a clear relationship seems to have been established only for antiphospholipid syndrome [1,6,19]; other conditions, such as inherited thrombophilia, are less commonly described and the real incidence in these cases seems to be different in several studies [13-18]. A possible explanation could also be related to an ethnic background and to the inclusion criteria of selected patients in the related studies. However, according to available data thrombophilia seems to be more frequent in young patients affected by RVO [28].

In this field, hyperhomocysteinemia has recently been identified as an emerging risk factor for RVO. Several reports in last few years, in fact, described frequently hyperhomocysteinemia in patients affected by RVO [29-34]. In the same reports the real incidence seems to differ not only for ethnical reasons but also for the difference in the criteria used for the inclusion. A relationship between hyperhomocysteinemia and RVO is well established for young subjects, but a lot of available studies focused on patients younger than 55 years [31,32]. Moreover, only a few studies on this topic enrolled more than 50 patients, such as the Blue Mountain Eye Study [28] and a meta-analysis by Cahill et al from US [30], this being a possible cause of underestimation of this issue. On the other hand, only few studies evaluated homocysteine metabolism in a more systematic way [31-35]. Although most studies focused, in fact, on MTHFR C677T gene polymorphism, not all researchers considered folate and vitamin B 12 serum levels.

In the present study we investigated homocysteinemia and MTHFR C677T gene polymorphism and other common variables associated to homocysteine metabolism, such as folate and vitamin B 12 serum levels.

We found data that patients affected by RVO had hyperhomocysteinemia, detected as fasting homocysteinemia and as post-load homocysteinemia, and the differences reached statistical significance (table 2). These data seem to be in agreement with those previously reported by other studies. However, this is the first report also showing total increase of homocysteinemia (i.e. Δ-Hcy) evaluated in patients affected by RVO and this difference also reached statistical significance (table 2).

Serum folate and vitamin B12 levels were also tested because strongly associated to homocysteine metabolism, in terms of a possible therapeutical role. Although serum folate and vitamin B12 levels were lower in patients compared also to control subjects, these differences did not reach statistical significance; however, also these data are in agreement with data reported by Yildirim et al on small population [33].

Yet, our data clearly show that hyperhomocysteinemia is more frequent than other common known risk factors for RVO, apart from hypertension and glaucoma (i.e. diabetes, dyslipidemia, smoking, hyperviscosity syndrome, vasculytis) (table 1). Only glaucoma and hypertension reached, in fact, statistical significance in patients affected by RVO versus control subjects in our study (table 1), confirming a frequent and an interesting possible pathophysiological role of homocysteine metabolism in RVO.

Moreover, the role of hyperhomocysteinemia in patients affected by RVO seems to be confirmed also by the high frequency of MTHFR C677T homozigosity in patients affected by RVO compared to control group (table 2). The TT genotype, in fact, has been associated more frequently to hyperhomocysteinemia, although this high incidence of TT genotype could be associated also to an ethnic background. This could be relevant because an epidemiological study written by Cappuccio et al underlined that TT genotype is more frequent in Caucasian subjects compared to Asian subjects [36] and a previous study by Cahill et al. [30] on subjects from US revealed that hyperhomocysteinemia but not TT genotype of MTHFR was a risk factor for RVO. So, although further studies on large based population should be performed also from a genetic point of view; our data suggest a prompt screening for homocysteine metabolism and MTHFR C677T gene polymorphism (in particular for Caucasian subjects) in patients affected by RVO. Further studied could also be addressed to understand also the role of another emerging MTHFR gene polymorphism (i.e. A1298C) sometime associated to hyperhomocysteinemia [37], alone and/or in association with MTHF C677T gene polymorphism, in large based population affected by RVO.

In conclusion, based on this study, we suggest a careful investigation on several metabolic parameters related to homocysteine metabolism in patients affected by RVO. A screening for hyperhomocysteinemia should be promptly performed particularly in patients without other common risk factors for RVO. These studies could have in fact a deep impact also on therapeutical aspects in order to better understand a possible role of folic acid and vitamin B12 fortification in the management of RVO.
